# *N*-(4-Acetyl­phen­yl)-4-meth­oxy­benzene­sulfonamide

**DOI:** 10.1107/S1600536813029875

**Published:** 2013-11-06

**Authors:** Thawanrat Kobkeatthawin, Suchada Chantrapromma, C. S. Chidan Kumar, Hoong-Kun Fun

**Affiliations:** a Department of Chemistry, Faculty of Science, Prince of Songkla University, Hat-Yai, Songkhla 90112, Thailand; b X-ray Crystallography Unit, School of Physics, Universiti Sains Malaysia, 11800 USM, Penang, Malaysia

## Abstract

The title compound, C_15_H_15_NO_4_S, was obtained by the condensation of 4-amino­aceto­phenone and 4-meth­oxy­benzene­sulfonyl chloride. The dihedral angle between the benzene rings is 86.56 (9)° and the mol­ecule has an approximate V-shaped conformation. The C atom of the meth­oxy group is roughly coplanar with its attached ring [deviation = 0.177 (3) Å], as is the methyl C atom of the acetyl group with its ring [deviation = 0.065 (2) Å]. An intra­molecular C—H⋯O inter­action generates an *S*(6) ring. In the crystal, N—H⋯O and C—H⋯O hydrogen bonds link the mol­ecules into [010] chains. Weak C—H⋯π inter­actions are also observed.

## Related literature

For related structures, see: Li *et al.* (2006 ▶); Xu *et al.* (2005 ▶). For background to and applications of sulfonamides, see: Alsughayer *et al.* (2011 ▶); Dragostin *et al.* (2013 ▶);
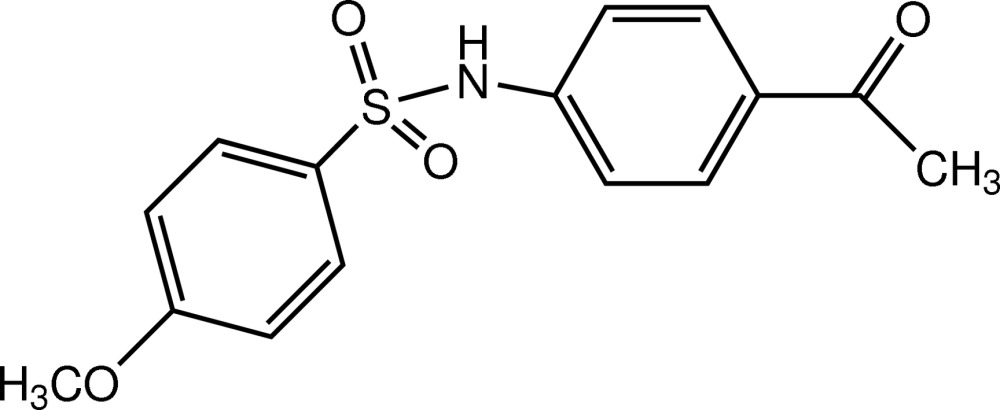


## Experimental

### 

#### Crystal data

C_15_H_15_NO_4_S
*M* *_r_* = 305.35
Monoclinic, 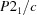

*a* = 12.8220 (3) Å
*b* = 8.2709 (2) Å
*c* = 14.6165 (4) Å
β = 112.841 (1)°
*V* = 1428.52 (6) Å^3^
*Z* = 4
Mo *K*α radiation
μ = 0.24 mm^−1^
*T* = 298 K
0.48 × 0.44 × 0.33 mm

#### Data collection

Bruker APEXII CCD diffractometer
Absorption correction: multi-scan (*SADABS*; Bruker, 2009 ▶) *T*_min_ = 0.894, *T*_max_ = 0.924
15571 measured reflections
4120 independent reflections
2593 reflections with *I* > 2σ(*I*)
*R*_int_ = 0.043

#### Refinement

*R*[*F* ^2^ > 2σ(*F* ^2^)] = 0.047
*wR*(*F* ^2^) = 0.135
*S* = 1.04
4120 reflections
196 parameters
H atoms treated by a mixture of independent and constrained refinement
Δρ_max_ = 0.20 e Å^−3^
Δρ_min_ = −0.34 e Å^−3^

### 

Data collection: *APEX2* (Bruker, 2009 ▶); cell refinement: *SAINT* (Bruker, 2009 ▶); data reduction: *SAINT*; program(s) used to solve structure: *SHELXTL* (Sheldrick, 2008 ▶); program(s) used to refine structure: *SHELXTL*; molecular graphics: *SHELXTL*; software used to prepare material for publication: *SHELXTL*, *PLATON* (Spek, 2009 ▶), *Mercury* (Macrae *et al.*, 2006 ▶) and *publCIF* (Westrip, 2010 ▶).

## Supplementary Material

Crystal structure: contains datablock(s) global, I. DOI: 10.1107/S1600536813029875/hb7153sup1.cif

Structure factors: contains datablock(s) I. DOI: 10.1107/S1600536813029875/hb7153Isup2.hkl

Supplementary material file. DOI: 10.1107/S1600536813029875/hb7153Isup3.cml

Additional supplementary materials:  crystallographic information; 3D view; checkCIF report

## Figures and Tables

**Table 1:** Hydrogen-bond geometry (Å, °) *Cg*1 and *Cg*2 are the centroids of the C1–C6 and C7–C12 rings, respectively.

*D*—H⋯*A*	*D*—H	H⋯*A*	*D*⋯*A*	*D*—H⋯*A*
N1—H1*N*1⋯O3^i^	0.85 (2)	2.05 (2)	2.896 (2)	172.3 (19)
C8—H8*A*⋯O2	0.93	2.38	3.030 (2)	127
C9—H9*A*⋯O1^ii^	0.93	2.53	3.459 (2)	174
C14—H14*A*⋯*Cg*1^i^	0.96	2.83	3.630 (3)	141
C14—H14*C*⋯*Cg*2^iii^	0.96	2.83	3.529 (2)	130
C15—H15*C*⋯*Cg*1^iv^	0.96	2.99	3.804 (3)	144

Symmetry codes: (i) 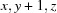; (ii) 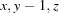; (iii) 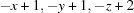; (iv) 
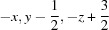
.

**Fig. 1.**
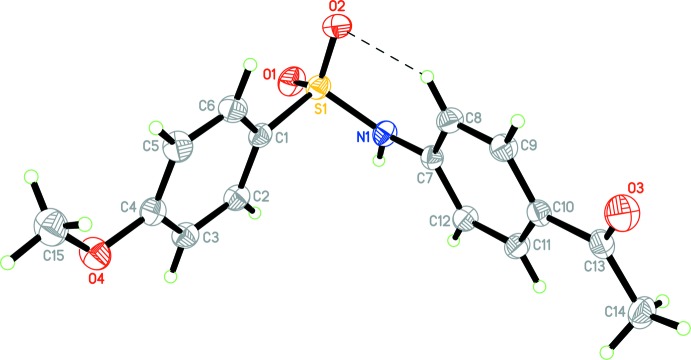
The molecular structure of (I), showing 40% probability displacement ellipsoids. The intramolecular C—H···O hydrogen bond is shown as a dashed line.

**Fig. 2.**
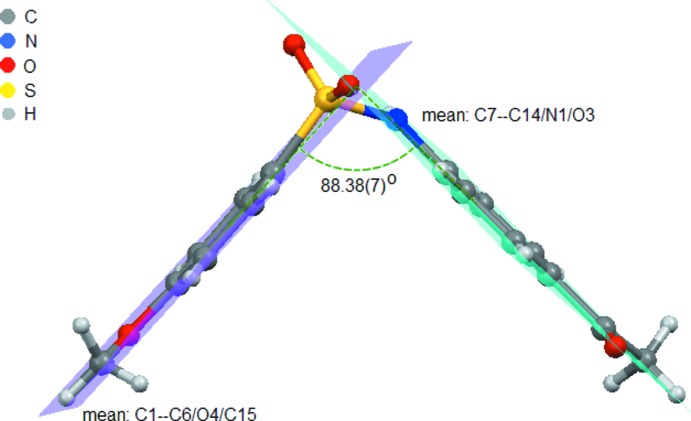
The V-shape conformation of the molecule.

**Fig. 3.**
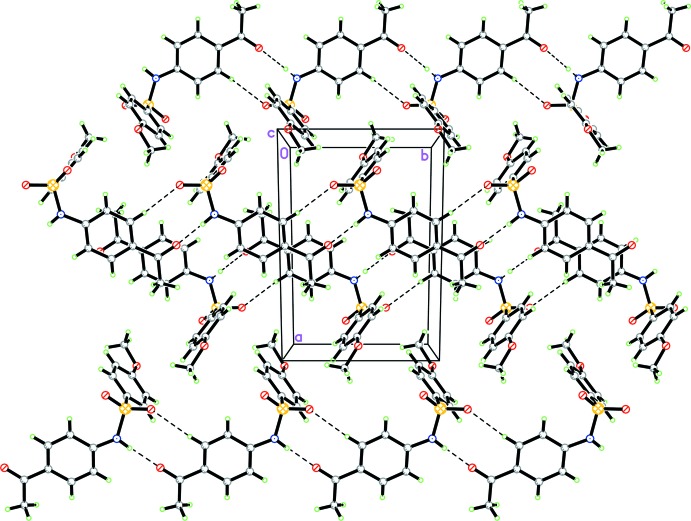
The crystal packing of the title compound viewed along the c axis. Hydrogen bonds were shown as dashed lines.

## supplementary crystallographic information

### 1. Comment

Sulfonamides containing an –SO_2_NH– group can be found in many pharmacologically active compounds: recent reports have described antibacterial (Alsughayer *et al.*, 2011) and antioxidant (Dragostin *et al.*, 2013) behaviour. As part of our ongoing research in this field, the title compound (I), a 4-methoxybenzene-sulfonamide derivative, was synthesized for being used as starting material for various syntheses. Herein the crystal structure of (I) is reported.

Figure 1 shows the molecular structure of (I), C_15_H_15_NO_4_S, suggesting a V-shaped conformation (Fig. 2). The benzene rings make the dihedral angle of 86.56 (9)°. The methoxy group is almost co-planar with its attached benzene ring with the deviation of 0.0310 (2) Å for the eight non H atoms (C1–C6/O4/C15) and the torsion angle C15–O4–C4–C5 = -2.2 (3)°. The amide group and acetyl substituent also lie in almost the same plane with the bound benzene ring with the deviation of 0.0220 (2) Å for the ten non H atoms (C7–C14/N1/O3) and the torsion angles of C11–C10–C13–O3 = -177.28 (18)° and C11–C10–C13–C14 = 2.6 (3)°. The dihedral angle between these two planes [C1–C6/O4/C15 and C7–C14/N1/O3] is 88.38 (7)° (Fig. 2). An intramolecular C8—H8A···O2 weak interaction generates an S(6) ring (Fig. 1) Bond distances of (I) are comparable with those in related structures (Li *et al.*, 2006 and Xu *et al.*, 2005).

In the crystal (Fig. 3), the molecules are linked by N—H···O hydrogen bonds and C—H···O weak interactions (Table 1) into chains along [010]. Weak C—H···π interactions are also observed (Table 1).

### 2. Experimental

The title compound was synthesized by condensation of 4-aminoacetophenone (0.40 g, 3 mmol) and 4-methoxybenzenesulfonyl chloride in CH_2_Cl_2_ (30 ml) in the presence of pyridine. The reaction mixture was refluxed for 24 hr at 40 °C and monitored with TLC for the completion of the reaction. Water was then added and the concoction was extracted with CH_2_Cl_2_. The solvent was evaporated under reduced pressure to yield the resulting solid of the title compound (yield 68%). Yellow blocks of (I) were recrystalized from acetone:CH_3_OH solution (1:1 *v*/*v*) by slow evaporation of the solvent at room temperature after several days, Mp. 448–449 K.

### 3. Refinement

Amide H atoms was located from the difference maps and refined isotropically. The remaining H atoms were positioned geometrically and allowed to ride on their parent atoms, with d(C—H) = 0.93 Å for aromatic and 0.96 for CH_3_ atoms. The *U*_iso_ values were constrained to be 1.5*U*_eq_ of the carrier atom for methyl H atoms and 1.2*U*_eq_ for the remaining H atoms. A rotating group model was used for the methyl groups.

### Crystal data

**Table:**

C_15_H_15_NO_4_S	*F*(000) = 640
*M**_r_* = 305.35	*D*_x_ = 1.420 Mg m^−^^3^
Monoclinic, *P*2_1_/*c*	Melting point = 448–449 K
Hall symbol: -P 2ybc	Mo *K*α radiation, λ = 0.71073 Å
*a* = 12.8220 (3) Å	Cell parameters from 4120 reflections
*b* = 8.2709 (2) Å	θ = 1.7–29.9°
*c* = 14.6165 (4) Å	µ = 0.24 mm^−^^1^
β = 112.841 (1)°	*T* = 298 K
*V* = 1428.52 (6) Å^3^	Block, yellow
*Z* = 4	0.48 × 0.44 × 0.33 mm

### Data collection

**Table:**

Bruker APEXII CCD diffractometer	4120 independent reflections
Radiation source: sealed tube	2593 reflections with *I* > 2σ(*I*)
Graphite monochromator	*R*_int_ = 0.043
φ and ω scans	θ_max_ = 29.9°, θ_min_ = 1.7°
Absorption correction: multi-scan (*SADABS*; Bruker, 2009)	*h* = −17→17
*T*_min_ = 0.894, *T*_max_ = 0.924	*k* = −11→11
15571 measured reflections	*l* = −20→19

### Refinement

**Table:**

Refinement on *F*^2^	Primary atom site location: structure-invariant direct methods
Least-squares matrix: full	Secondary atom site location: difference Fourier map
*R*[*F*^2^ > 2σ(*F*^2^)] = 0.047	Hydrogen site location: inferred from neighbouring sites
*wR*(*F*^2^) = 0.135	H atoms treated by a mixture of independent and constrained refinement
*S* = 1.04	*w* = 1/[σ^2^(*F*_o_^2^) + (0.0625*P*)^2^ + 0.0913*P*] where *P* = (*F*_o_^2^ + 2*F*_c_^2^)/3
4120 reflections	(Δ/σ)_max_ = 0.001
196 parameters	Δρ_max_ = 0.20 e Å^−^^3^
0 restraints	Δρ_min_ = −0.34 e Å^−^^3^

### Special details

**Table:**

Geometry. All e.s.d.'s (except the e.s.d. in the dihedral angle between two l.s. planes) are estimated using the full covariance matrix. The cell e.s.d.'s are taken into account individually in the estimation of e.s.d.'s in distances, angles and torsion angles; correlations between e.s.d.'s in cell parameters are only used when they are defined by crystal symmetry. An approximate (isotropic) treatment of cell e.s.d.'s is used for estimating e.s.d.'s involving l.s. planes.
Refinement. Refinement of *F*^2^ against ALL reflections. The weighted *R*-factor *wR* and goodness of fit *S* are based on *F*^2^, conventional *R*-factors *R* are based on *F*, with *F* set to zero for negative *F*^2^. The threshold expression of *F*^2^ > σ(*F*^2^) is used only for calculating *R*-factors(gt) *etc*. and is not relevant to the choice of reflections for refinement. *R*-factors based on *F*^2^ are statistically about twice as large as those based on *F*, and *R*- factors based on ALL data will be even larger.

### Fractional atomic coordinates and isotropic or equivalent isotropic displacement parameters (Å^2^)

**Table:**

	*x*	*y*	*z*	*U*_iso_*/*U*_eq_	
S1	0.21179 (4)	1.00630 (5)	1.08283 (4)	0.04186 (15)	
O1	0.21200 (12)	1.17413 (16)	1.10664 (11)	0.0546 (4)	
O2	0.14687 (11)	0.89569 (17)	1.11408 (11)	0.0529 (4)	
O3	0.48523 (14)	0.21499 (16)	1.11408 (12)	0.0624 (4)	
O4	0.05688 (12)	0.98389 (16)	0.64850 (11)	0.0540 (4)	
N1	0.34483 (13)	0.95337 (19)	1.13298 (12)	0.0408 (4)	
C1	0.16988 (14)	0.9867 (2)	0.95362 (14)	0.0378 (4)	
C2	0.22355 (15)	1.0787 (2)	0.90451 (14)	0.0418 (4)	
H2A	0.2849	1.1439	0.9404	0.050*	
C3	0.18492 (15)	1.0722 (2)	0.80272 (15)	0.0432 (4)	
H3A	0.2209	1.1321	0.7697	0.052*	
C4	0.09193 (16)	0.9760 (2)	0.74878 (14)	0.0415 (4)	
C5	0.04067 (17)	0.8825 (2)	0.79829 (15)	0.0487 (5)	
H5A	−0.0201	0.8161	0.7627	0.058*	
C6	0.07979 (16)	0.8882 (2)	0.90017 (15)	0.0466 (5)	
H6A	0.0454	0.8254	0.9333	0.056*	
C7	0.38891 (14)	0.7983 (2)	1.12776 (12)	0.0353 (4)	
C8	0.32855 (16)	0.6554 (2)	1.12191 (14)	0.0422 (4)	
H8A	0.2549	0.6591	1.1191	0.051*	
C9	0.37892 (16)	0.5091 (2)	1.12029 (15)	0.0427 (4)	
H9A	0.3381	0.4144	1.1157	0.051*	
C10	0.48954 (16)	0.4993 (2)	1.12535 (13)	0.0381 (4)	
C11	0.54871 (16)	0.6432 (2)	1.13233 (14)	0.0422 (4)	
H11A	0.6230	0.6394	1.1368	0.051*	
C12	0.49943 (15)	0.7902 (2)	1.13275 (14)	0.0413 (4)	
H12A	0.5400	0.8849	1.1364	0.050*	
C13	0.54079 (17)	0.3380 (2)	1.12393 (14)	0.0427 (4)	
C14	0.66124 (18)	0.3279 (3)	1.13447 (15)	0.0542 (5)	
H14A	0.6814	0.2168	1.1316	0.081*	
H14B	0.7094	0.3736	1.1970	0.081*	
H14C	0.6704	0.3870	1.0815	0.081*	
C15	−0.0427 (2)	0.8943 (3)	0.59083 (17)	0.0666 (6)	
H15A	−0.0602	0.9117	0.5215	0.100*	
H15B	−0.1050	0.9300	0.6067	0.100*	
H15C	−0.0297	0.7812	0.6057	0.100*	
H1N1	0.3889 (18)	1.031 (2)	1.1334 (15)	0.047 (6)*	

### Atomic displacement parameters (Å^2^)

**Table:**

	*U*^11^	*U*^22^	*U*^33^	*U*^12^	*U*^13^	*U*^23^
S1	0.0414 (2)	0.0396 (3)	0.0475 (3)	0.00720 (19)	0.0204 (2)	−0.00056 (19)
O1	0.0597 (9)	0.0422 (8)	0.0628 (9)	0.0125 (6)	0.0248 (7)	−0.0084 (6)
O2	0.0485 (8)	0.0588 (9)	0.0603 (9)	0.0041 (6)	0.0309 (7)	0.0070 (7)
O3	0.0769 (10)	0.0329 (7)	0.0842 (11)	−0.0013 (7)	0.0386 (9)	0.0008 (7)
O4	0.0561 (9)	0.0552 (9)	0.0464 (9)	−0.0140 (7)	0.0152 (7)	−0.0038 (6)
N1	0.0411 (8)	0.0332 (8)	0.0468 (10)	0.0015 (7)	0.0156 (7)	−0.0022 (7)
C1	0.0337 (8)	0.0337 (9)	0.0464 (10)	0.0046 (7)	0.0158 (8)	0.0028 (7)
C2	0.0365 (9)	0.0369 (10)	0.0508 (12)	−0.0058 (7)	0.0156 (8)	−0.0021 (8)
C3	0.0404 (10)	0.0378 (10)	0.0528 (12)	−0.0039 (8)	0.0196 (9)	0.0016 (8)
C4	0.0412 (9)	0.0374 (10)	0.0438 (11)	0.0011 (8)	0.0143 (8)	−0.0015 (8)
C5	0.0444 (10)	0.0437 (11)	0.0526 (12)	−0.0126 (8)	0.0130 (9)	−0.0018 (9)
C6	0.0435 (10)	0.0425 (10)	0.0552 (12)	−0.0073 (8)	0.0209 (9)	0.0039 (9)
C7	0.0381 (8)	0.0344 (9)	0.0321 (9)	0.0022 (7)	0.0121 (7)	−0.0007 (7)
C8	0.0385 (9)	0.0405 (10)	0.0496 (11)	−0.0001 (8)	0.0193 (8)	0.0004 (8)
C9	0.0455 (10)	0.0337 (9)	0.0513 (11)	−0.0035 (8)	0.0213 (9)	0.0010 (8)
C10	0.0440 (9)	0.0346 (9)	0.0356 (9)	0.0016 (7)	0.0153 (8)	0.0026 (7)
C11	0.0373 (9)	0.0411 (10)	0.0494 (11)	0.0022 (8)	0.0179 (8)	0.0020 (8)
C12	0.0391 (9)	0.0346 (9)	0.0506 (11)	−0.0030 (7)	0.0180 (8)	0.0004 (8)
C13	0.0552 (11)	0.0377 (10)	0.0356 (10)	0.0069 (8)	0.0180 (9)	0.0038 (7)
C14	0.0590 (12)	0.0497 (12)	0.0530 (13)	0.0166 (10)	0.0208 (10)	−0.0003 (9)
C15	0.0587 (14)	0.0790 (16)	0.0519 (14)	−0.0175 (12)	0.0103 (11)	−0.0074 (12)

### Geometric parameters (Å, º)

**Table:**

S1—O2	1.4261 (14)	C7—C12	1.392 (2)
S1—O1	1.4308 (13)	C7—C8	1.397 (2)
S1—N1	1.6335 (16)	C8—C9	1.376 (2)
S1—C1	1.7590 (19)	C8—H8A	0.9300
O3—C13	1.218 (2)	C9—C10	1.394 (3)
O4—C4	1.358 (2)	C9—H9A	0.9300
O4—C15	1.433 (3)	C10—C11	1.394 (2)
N1—C7	1.416 (2)	C10—C13	1.491 (2)
N1—H1N1	0.85 (2)	C11—C12	1.371 (2)
C1—C6	1.381 (3)	C11—H11A	0.9300
C1—C2	1.397 (3)	C12—H12A	0.9300
C2—C3	1.375 (3)	C13—C14	1.494 (3)
C2—H2A	0.9300	C14—H14A	0.9600
C3—C4	1.395 (3)	C14—H14B	0.9600
C3—H3A	0.9300	C14—H14C	0.9600
C4—C5	1.387 (3)	C15—H15A	0.9600
C5—C6	1.375 (3)	C15—H15B	0.9600
C5—H5A	0.9300	C15—H15C	0.9600
C6—H6A	0.9300		
			
O2—S1—O1	119.39 (9)	C9—C8—C7	119.50 (17)
O2—S1—N1	108.79 (8)	C9—C8—H8A	120.3
O1—S1—N1	104.35 (9)	C7—C8—H8A	120.3
O2—S1—C1	108.17 (9)	C8—C9—C10	121.67 (17)
O1—S1—C1	108.75 (8)	C8—C9—H9A	119.2
N1—S1—C1	106.72 (8)	C10—C9—H9A	119.2
C4—O4—C15	117.12 (16)	C11—C10—C9	117.90 (16)
C7—N1—S1	126.00 (13)	C11—C10—C13	122.34 (17)
C7—N1—H1N1	113.8 (14)	C9—C10—C13	119.76 (16)
S1—N1—H1N1	112.0 (14)	C12—C11—C10	121.26 (17)
C6—C1—C2	120.09 (18)	C12—C11—H11A	119.4
C6—C1—S1	120.10 (15)	C10—C11—H11A	119.4
C2—C1—S1	119.69 (14)	C11—C12—C7	120.25 (16)
C3—C2—C1	119.49 (17)	C11—C12—H12A	119.9
C3—C2—H2A	120.3	C7—C12—H12A	119.9
C1—C2—H2A	120.3	O3—C13—C10	120.55 (18)
C2—C3—C4	120.26 (18)	O3—C13—C14	119.98 (17)
C2—C3—H3A	119.9	C10—C13—C14	119.47 (17)
C4—C3—H3A	119.9	C13—C14—H14A	109.5
O4—C4—C5	124.41 (17)	C13—C14—H14B	109.5
O4—C4—C3	115.76 (17)	H14A—C14—H14B	109.5
C5—C4—C3	119.82 (18)	C13—C14—H14C	109.5
C6—C5—C4	119.90 (18)	H14A—C14—H14C	109.5
C6—C5—H5A	120.0	H14B—C14—H14C	109.5
C4—C5—H5A	120.0	O4—C15—H15A	109.5
C5—C6—C1	120.40 (18)	O4—C15—H15B	109.5
C5—C6—H6A	119.8	H15A—C15—H15B	109.5
C1—C6—H6A	119.8	O4—C15—H15C	109.5
C12—C7—C8	119.41 (16)	H15A—C15—H15C	109.5
C12—C7—N1	117.40 (16)	H15B—C15—H15C	109.5
C8—C7—N1	123.14 (17)		
			
O2—S1—N1—C7	53.22 (18)	C2—C1—C6—C5	−1.4 (3)
O1—S1—N1—C7	−178.31 (15)	S1—C1—C6—C5	174.65 (15)
C1—S1—N1—C7	−63.27 (17)	S1—N1—C7—C12	151.37 (15)
O2—S1—C1—C6	5.98 (17)	S1—N1—C7—C8	−31.2 (3)
O1—S1—C1—C6	−125.08 (15)	C12—C7—C8—C9	−0.6 (3)
N1—S1—C1—C6	122.88 (15)	N1—C7—C8—C9	−177.92 (17)
O2—S1—C1—C2	−177.94 (13)	C7—C8—C9—C10	0.6 (3)
O1—S1—C1—C2	51.00 (16)	C8—C9—C10—C11	0.1 (3)
N1—S1—C1—C2	−61.03 (15)	C8—C9—C10—C13	179.65 (17)
C6—C1—C2—C3	1.0 (3)	C9—C10—C11—C12	−1.0 (3)
S1—C1—C2—C3	−175.13 (14)	C13—C10—C11—C12	179.50 (17)
C1—C2—C3—C4	0.8 (3)	C10—C11—C12—C7	1.0 (3)
C15—O4—C4—C5	2.7 (3)	C8—C7—C12—C11	−0.3 (3)
C15—O4—C4—C3	−176.22 (18)	N1—C7—C12—C11	177.24 (17)
C2—C3—C4—O4	176.85 (16)	C11—C10—C13—O3	−177.28 (18)
C2—C3—C4—C5	−2.2 (3)	C9—C10—C13—O3	3.2 (3)
O4—C4—C5—C6	−177.21 (18)	C11—C10—C13—C14	2.6 (3)
C3—C4—C5—C6	1.7 (3)	C9—C10—C13—C14	−176.96 (18)
C4—C5—C6—C1	0.1 (3)		

### Hydrogen-bond geometry (Å, º)

Cg1 and Cg2 are the centroids of the C1–C6 and C7–C12 rings, respectively.

**Table:**

*D*—H···*A*	*D*—H	H···*A*	*D*···*A*	*D*—H···*A*
N1—H1*N*1···O3^i^	0.85 (2)	2.05 (2)	2.896 (2)	172.3 (19)
C8—H8*A*···O2	0.93	2.38	3.030 (2)	127
C9—H9*A*···O1^ii^	0.93	2.53	3.459 (2)	174
C14—H14*A*···*Cg*1^i^	0.96	2.83	3.630 (3)	141
C14—H14*C*···*Cg*2^iii^	0.96	2.83	3.529 (2)	130
C15—H15*C*···*Cg*1^iv^	0.96	2.99	3.804 (3)	144

Symmetry codes: (i) *x*, *y*+1, *z*; (ii) *x*, *y*−1, *z*; (iii) −*x*+1, −*y*+1, −*z*+2; (iv) −*x*, *y*−1/2, −*z*+3/2.
